# A Novel RANKL-Targeted Furoquinoline Alkaloid Ameliorates Bone Loss in Ovariectomized Osteoporosis through Inhibiting the NF-*κ*B Signal Pathway and Reducing Reactive Oxygen Species

**DOI:** 10.1155/2022/5982014

**Published:** 2022-11-04

**Authors:** Puiian Wong, Zheng Lv, Jinglan Li, Qiushi Wei, LiangLiang Xu, Bin Fang, Yiwen Luo, Mincong He

**Affiliations:** ^1^The Third Clinical Medical School, Guangzhou University of Chinese Medicine, Guangzhou, 510405 Guangdong, China; ^2^The First Clinical Medical School, Guangzhou University of Chinese Medicine, Guangzhou, 510405 Guangdong, China; ^3^The Third Affiliated Hospital of Guangzhou University of Chinese Medicine, Guangzhou, 510240 Guangdong, China; ^4^Guangdong Research Institute for Orthopedics and Traumatology of Chinese Medicine, Guangzhou, China; ^5^Lingnan Medical Research Center of Guangzhou University of Chinese Medicine, China; ^6^The First Affiliated Hospital of Guangzhou University of Chinese Medicine, Guangzhou, 510405 Guangdong, China

## Abstract

Dysregulation of osteoclast-osteoblast balance, resulting in abnormal bone remodeling, is responsible for postmenopausal osteoporosis (PMOP) or other secondary forms of osteoporosis. We demonstrated that dictamnine (DIC), a novel RANKL-targeted furoquinoline alkaloid, inhibits osteoclastogenesis by facilitating the activities of reactive oxygen species (ROS), NF-*κ*B, and NFATc1 in vitro and prevents the development of OVX-induced osteoporosis mouse models in vivo. *Methods*. The docking mechanism of DIC and RANKL was initially identified by protein–ligand molecular docking. RNA sequencing was performed and analyzed to reveal the potential mechanism and signaling pathway of the antiosteoporosis effects of DIC. To verify the sequencing results, we examined the impact of DIC on RANKL-induced osteoclast differentiation, bone resorption, F-actin ring production, ROS generation, and NF-*κ*B activation in osteoclasts in vitro. Moreover, a luciferase assay was performed to determine the binding and transcriptional activity of Nrf2 and NF-*κ*B. The *in vivo* efficacy of DIC was assessed with an ovariectomy- (OVX-) induced osteoporosis model, which was analyzed using micro-CT and bone histomorphometry. *Results*. The molecular docking results indicated that DIC could bind particularly to RANKL. RNA-seq confirmed that DIC could regulate the osteoclast-related pathway. DIC suppressed osteoclastogenesis, bone resorption, F-actin belt formation, osteoclast-specific gene expression, and ROS activity by preventing NFATc1 expression and affecting NF-*κ*B signaling pathways in vitro. The luciferase assay showed that DIC not only suppressed the activity of Nrf2 but also contributed to the combination of Nrf2 and NF-*κ*B. Our *in vivo* study indicated that DIC protects against OVX-induced osteoporosis and preserves bone volume by inhibiting osteoclast activity and function. *Conclusions*. DIC can ameliorate osteoclast formation and OVX-induced osteoporosis and therefore is a potential therapeutic treatment for osteoporosis.

## 1. Introduction

Osteoporosis is a severe disease that is characterized by the destruction of the bone microstructure, high disability, and mortality, bringing a heavy financial burden [1]. According to statistics, females have a higher incidence of osteoporosis than males [2]. Postmenopausal females are more susceptible to losing bone mass because of estrogen deficiency [3]. Estrogen is the key to maintaining bone mass. When menopause begins, the body goes through 4 to 8 years of bone loss acceleration; then, the loss becomes more gradual [4]. Brittle fracture leading to high mortality of PMOP is the most grievous complication of osteoporosis [5].

The balance between osteoblasts and osteoclasts is the key to maintaining bone mass [6]. Osteoblasts are responsible for bone formation, while osteoclasts are responsible for resorption. However, dysregulation of this balance results in abnormal bone remodeling, leading to both PMOP and secondary osteoporosis [7, 8]. The ancestors of osteoclasts are mononuclear macrophage lineage cells, which can fuse to multinucleated cells called osteoclasts. M-CSF (macrophage colony-stimulating factor) and RANKL (receptor activator of nuclear factor-*κ*B ligand) are necessary for this biological process. RANKL and its receptor RANK activate the master regulator of osteoclastogenesis, NFATc1, and CTSK, to induce the expression of osteoclastogenesis-related genes [9]. Ha et al. found that ROS are a medium in the signaling pathways and cellular events induced by RANKL [10].

The prevention and treatment of osteoporosis remain crucial. Therefore, derived molecules and natural products are essential in developing new therapies for osteoporosis [11]. Dictamnine (DIC), a kind of furoquinoline alkaloid, comes from the Chinese herbal medicine root bark of the family Rutaceae perennial herbs—Dictamnus dasycarpus [12]. It has antifungal [13], antiangiogenic, and anticancer anti-inflammatory properties [11]. According to the latest research, Lin et al. demonstrated that dictamnine can efficiently scavenge reactive oxygen species (ROS) and mitochondrial ROS (mROS) and reduce interleukin-1*β* (IL-1*β*) and tumor necrosis factor-*α* (TNF-*α*) expression, NLRP3 inflammasome activation, and NF-*κ*B expression [14]. Therefore, DIC is a potential drug with significant value for developing new therapeutics. However, the role of DIC in osteoporosis has not been studied. We hypothesized that DIC could suppress osteoclasts and thus prevent PMOP.

## 2. Materials and Methods

### 2.1. Drug Preparation

The standard for dictamnine was purchased from a company specializing in preparing traditional Chinese medicine monomers (Weikeqi, China), diluted in DMSO, and stored at −20°C. We used different concentrations (50 *μ*m, 75 *μ*m, 100 *μ*m, and 150 *μ*m) of dictamnine for further experiments [15].

### 2.2. Cell Viability Assay

To measure whole-cell viability after DIC induction at two different time points (24 hrs, 48 hrs), we measured cell viability by setting up four different concentrations of DIC. RAW264.7 cells were plated in 96-well plates at a density of 5000 cells per well. After the intervention of DIC at 24 or 48 hours, the cell viability was measured by the CCK8 method and the absorbance was measured by Multiskan Spectrum 1 hour after adding 10 *μ*l CCK8 reagent (Fdbio, China) to each well. The experiment will test the effects of different concentrations of DIC on cell viability to ensure that the DIC's effect on further osteoclast differentiation is not caused by inducing cell death and to select the appropriate concentration for subsequent experiments.

### 2.3. Osteoclast Differentiation

RAW264.7 cells were used to measure the ability of osteoclasts to differentiate following dictamnine administration. Cells were passaged after recovery; the experiment was carried out after their state was stable. The cells were cultured in *α*-MEM (HyClone, USA) containing 5% fetal bovine serum (Excell Bio, China) and 1% penicillin/streptomycin in a cell incubator containing 5% carbon dioxide. TRAP staining was used to measure mature multinuclear osteoclasts; 3000 cells per well were seeded in 96-well plates with 50 ng/ml RANKL induction (BD, USA), and the induction medium was replaced every two days until multinuclear osteoclasts were produced [16]. The cells were fixed with 4% PFA for 30 minutes and washed with PBS 3 times, and TRAP staining solution (Sigma, USA) was added and incubated at 37°C for 1 hour. The images were captured in three areas randomly by a microscope; ImageJ was used for quantitative analysis.

### 2.4. Protein–Ligand Molecular Docking

Dictamnine's 2D structures and the target RANKL-RANK complex crystal structure were downloaded from the PubChem and PDB databases (ID: 4GIQ). The latter selects a model with ligand binding smaller than 3 Å. The crystal structure was imported into PyMOL 1.7.2.1 software (https://pymol.org/2/) for the dehydration, hydrogenation, and separation of ligands. AutoDockTools 1.5.6 was used to construct the docking grid of RANKL [17, 18]. Docking was completed by AutoDock software. The lowest binding energy in the docking conformation was selected to observe the binding effect by matching the ligand and intermolecular interactions (hydrophobicity, etc.).

### 2.5. RNA Sequencing

RAW 264.7 cells that experienced two days of RANKL stimulation were cultured with DIC (150 *μ*m) compared with the control group. Total RNA was harvested on day 2 of the culture. Cells from the DIC (*n* = 3) and control (*n* = 3) groups were homogenized in TRIzol reagent for RNA extraction. A total amount of 1 *μ*g RNA per sample was used as input material for the RNA sample preparations. Sequencing libraries were generated for Illumina. According to the manufacturer's instructions, a PE Cluster Kit CBOT-HS (Illumina) was used to complete the clustering of index-coded samples on the BotCluster generation system. The library preparation was sequenced on the Illumina HiSeq platform, and paired terminal readings were produced. The filtered assignments were aligned with the Mus musculus genome (mus_musculus.grcm38) using HISAT2 (version 2.0.5). HTSeq (version 0.6.1) was used to calculate the mapping reading for each gene. Differential expression analysis between the 2 groups was performed using the DESeq2 R package (version 1.20.0). Expression of which |fold change (FC)| ≥ 1.2 and *P* < 0.05 were screened out as significantly differentially expressed genes.

### 2.6. GO and KEGG Enrichment Analysis and Protein–Protein Interaction (PPI) Network Construction

Functional enrichment analysis was performed on the differentially expressed genes. Gene Ontology (GO) (database last updated 22 February 2020) and Kyoto Encyclopedia of Genes and Genomes (KEGG) (last updated 14 January 2020) pathway annotations were downloaded. The downloaded data were used to classify significantly differentially expressed genes in specific GO terms and KEGG pathways. Only significantly enriched KEGG functional categories (*P* < 0.05) are depicted according to their *P* values (−log10 (*P* value)). KOBAS software (version 3.0) was used for KEGG pathway enrichment analysis. Pathway *P*adj values less than 0.05 were considered to be significantly enriched. The string obtained the PPI network from the downregulated genes extracted from the differentially expressed genes. The minimum required interaction score was set as high confidence (0.700). Disconnected nodes in the network were removed.

### 2.7. qRT–PCR

Osteoclastogenesis-specific gene expression was measured by qRT–PCR. RAW264.7 cells were seeded in 48-well plates and simultaneously subjected to 2 days of RANKL induction and DIC intervention. Total RNA was extracted by NucleoZOL (Macherey Nagel, MN, Germany) and centrifuged 3 times to separate the RNA and protein, and the RNA was purified with 75% alcohol. A NanoDrop2000 (Thermo, USA) was used to measure the concentration of RNA. RNA was reverse transcribed into cDNA by reverse transcriptase (AG, China) with a total specimen volume of 10 *μ*l. A Bio-Rad PCR instrument was used for the PCR experiment. The primers were designed as shown in Table 1.

### 2.8. Western Blot

Cells were seeded in 25 cm^2^ culture dishes, and DIC intervention was performed for 3 or 5 days or for 20 or 60 min. The cells were then lysed in a mixture containing RIPA and PMSF (1 : 100) and phosphatase inhibitors (1 : 100). The suspension was incubated in EP tubes for 30 min and centrifuged for 20 min at 12000 rpm at 4°C. The supernatant was removed, and the concentration was measured using a Bradford assay kit (Solarbio, China). The sample specimen was prepared with 5x loading buffer. After making the running gels, the samples ran in the upper gel at 80 V for 20 minutes; then, the samples ran in the lower gel at 100 V for 80 minutes. PVDF membranes were transformed at 55 V for 75 min. The PVDF membranes were blocked in QuickBlock Western buffer (Beyotime, China) and incubated with the primary antibodies (1 : 1000) at 4°C overnight. The next day, secondary antibodies (1 : 5000) were incubated and washed in TBST buffer and then developed by ECL solution (Beyotime, China).

### 2.9. F-Actin Belt

Fresh RAW264.7 cells were plated in 96-well plates, and the cell density was adjusted to 3000 cells per well. The osteoclast induction process is described above. Phalloidin-TRITC powder diluted in sterile water was prepared as a storage buffer. The storage solution was diluted with PBS at ratios of 1 : 40–1 : 200. The cells were washed 3 times with PBS, fixed with 4% PFA on ice for 15 min, and washed with PBS again. The cells were then permeabilized in 0.5% Triton X-100 PBS buffer at room temperature. Then, 70 *μ*l of rhodamine-phalloidin storage solution was added to each well (96-well plate) and incubated at room temperature for 20 min away from light. Finally, a DAPI antifluorescence quenching agent (Beyotime, China) was added. A fluorescence microscope was used for observation [19]. The nucleus number and F-actin belt size were measured by using ImageJ software.

## 3. Reactive Oxygen Species Level Determination

The intervention of RAW264.7 cells is described as follows. Cells were stimulated with 3% hydrogen peroxide to keep the peroxide level stable and were set as a positive control. H2DCFDA (MCE, China) is a cell-permeable probe for the detection of intracellular reactive oxygen species (ROS) [20]. H2DCFDA was dissolved to a specified concentration according to the manufacturer's instructions. After stimulation with 3% hydrogen peroxide for 24 h, the medium was removed and the H2DCFDA probe at the specified concentration was added, avoiding light, and incubated for 30 min. Fluorescence microscopy and flow cytometry were performed simultaneously [21]. The quantitative analyses of mean fluorescence intensity are presented using ImageJ software.

### 3.1. Luciferase Reporter Assay of Nrf2 and NF-*κ*B

The RAW264.7-cell line was stably transfected with NF-*κ*B [22]. The luciferase reporter assay was performed using the dual-luciferase reporter assay system (Promega, Madison, WI, USA) according to the manufacturer's instructions. In brief, GPL4-Basic and GPL4-Nrf2 were transfected into RAW264.7 cells with NF-*κ*B using Lipofectamine 2000 (Invitrogen). Cells were cultured overnight and then pretreated with DIC and stimulated with RANKL for 48 hours. Luciferase activity was measured using a GloMaxTM 20/20 single-tube luminometer (Promega, Madison, WI, USA).

### 3.2. OVX-Mouse Model Establishment

Twenty-four C57BL/J female mice were purchased from the Laboratory Animal Center, Guangzhou University of Traditional Chinese Medicine (Guangzhou, China). Their age was approximately 8 weeks. These animals received ovariectomy at 12 weeks. The mice were divided into 4 groups: sham group, OVX group, OVX + DIC (10 mg/kg) group, and OVX + DIC (20 mg/kg) group; each group contained six mice. In the sham group, only the skin was cut without ovariectomy. After the surgery, every mouse was injected with penicillin to avoid infection.

For drug administration, 20 mg dictamnine (Weikeqi, China) was diluted in 500 *μ*l DMSO to a final concentration of 40 mg/ml. Then, the dictamnine was diluted with PBS and DMSO at 10 mg/kg or 20 mg/kg as low or high concentrations. The sham and OVX groups were treated with DMSO and PBS as negative and positive controls, respectively. The whole process of modeling and drug administration continued for 6 weeks. The mice were sacrificed and eviscerated, and their limbs, especially those of the articulatio genus, were fixed with neutral formalin for two days, which was then replaced with 75% alcohol for further experiments.

### 3.3. Micro-CT Scan and Analysis

After replacing the formalin with 75% alcohol, the limbs were wrapped in a cylindrical tube to fix the position. A SkyScan 1176 micro-CT scanner (Bruker micro-CT, Kontich, Belgium) was used for scanning. The scanning was carried out using the following settings: voltage 60 kV, source current 417 *μ*A, Al 0.5 mm filter, pixel size 9 *μ*m, and rotation step 0.5 degrees. A region of interest (0.009 mm below the growth plate on the distal tibia with a height of 0.27 mm) was selected for trabecular bone analysis. The bone mineral density (BMD), trabecular separation (Tb.Sp), trabecular thickness (Tb.Th), bone volume/tissue volume (BV/TV), trabeculae (Tb.N), and trabecular pattern factor (Tb.Pf) were measured by a CT Analyzer program (Bruker micro-CT, Kontich, Belgium). Two- and three-dimensional images were generated using DataViewer and CTvol software (Bruker micro-CT, Kontich, Belgium), respectively.

### 3.4. Bone Histomorphometry, HE Staining, Tartrate-Resistant Acid Phosphatase (TRAP) Staining, and Immunocytochemistry

Osseous tissue was steeped in 9% formic acid for decalcification. After two weeks, a 1 ml syringe needle was used to test the degree of decalcification; when the needle passed easily through the tissue, the tissue was determined to be dehydrated. The tissues were embedded in wax and cut into 5 *μ*m thick paraffin sections. The paraffin sections were exposed to dryers to melt the wax, and xylene and gradient alcohol were utilized to dewax and rehydrate them for further histological staining. For HE staining, hematoxylin-eosin one-step staining solution (Solarbio, China) was utilized for 10 minutes. Hydrochloric acid alcohol was used for differentiation. For IHC staining, 3% hydrogen peroxide inactivated catalase activity and sodium citrate antigen repair solution was used for antigen repair. The cells were sealed with a sealing solution containing Triton X-100 and FBS for an hour and then incubated with the primary antibodies. After setting the secondary antibody for the following day, DAB solution was utilized to color the sections. Finally, the nuclei were counterstained with hematoxylin (Biosharp, China). TRAP staining was performed using a commercial staining kit (Sigma, St. Louis, MO, USA) following the manufacturer's instructions. Bone histomorphometric analysis was performed using BIOQUANT OSTEO software (BIOQUANT Image Analysis Corporation, Nashville, TN, USA). Quantitative analyses of the mean OD value of IHC were performed using ImageJ software.

### 3.5. Statistical Analysis

All statistical data were graphed and analyzed by GraphPad Prism software version 6 (GraphPad Software, San Diego, CA, USA). Differences between groups were compared using one-way ANOVA. *P* < 0.5 indicated statistical significance.

## 4. Results

### 4.1. Combination of the Dictamnine-RANKL Complex

**Figure 1:**
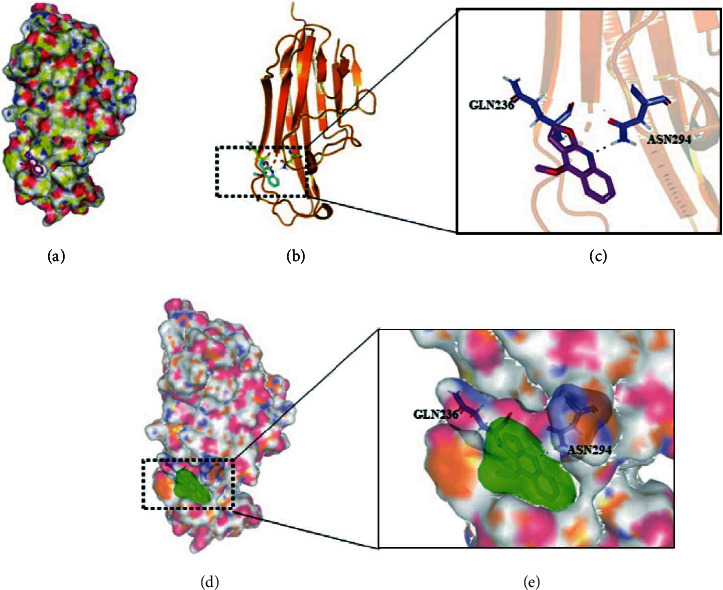
Establishment of the RANKL crystal model. (a) A potential binding site for DIC in a hydrophobic domain containing hydrogen-bond accepter and donor was predicted. (b, c) The binding site for Rob docking began at residues GLN236 and extended toward ASN294. (d, e) A strong affinity between DIC and RANKL was observed in computational docking.

A crystal model of RANKL was constructed as a basic template for DIC matching (Figure 1(a)). A potential binding spot for DIC in a hydrophobic domain containing a hydrogen-bond accepter and donor was predicted (Figures 1(b) and 1(c)). The critical area for DIC docking began at residues GLN236 and extended toward ASN294 (Figures 1(d) and 1(e)). A strong affinity between DIC and RANKL was observed in computational docking (binding free energy = 5.23 kcal/mol). Our results generally reported that the strong relationship between DIC and RANKL was based on their complex noncovalent interactions.

### 4.2. Differential Expression Analysis in the DIC-Treated and Control Groups

**Figure 2:**
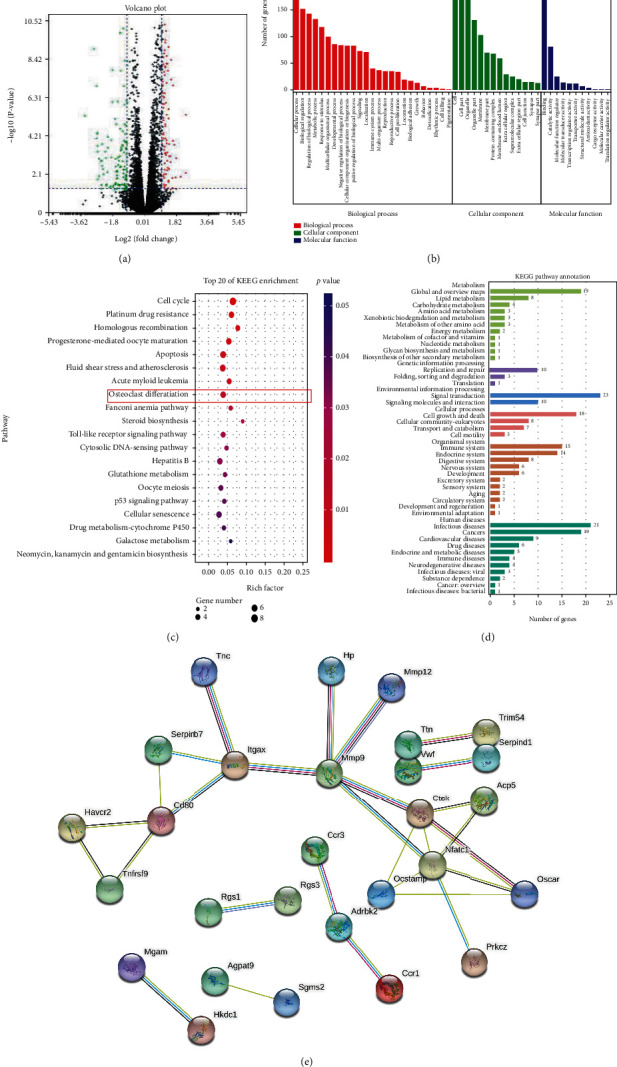
A total of 139 upregulated genes and 420 downregulated genes were identified in RNA-seq (a). GO enrichment analysis based on biological processes (BPs), molecular functions (MFs), and cellular components (CCs) was performed to gain insights into the physical part (b). Furthermore, KEGG pathway analysis was performed to identify the top 20 signaling pathways affected by differentially expressed genes (c). KEGG pathway annotation indicated that multiple genes were enriched in signal transduction and signaling molecules and interactions, confirming the KEGG enrichment findings (d). A PPI network of coregulated downregulated genes was obtained and visualized (e).

RNA sequencing analysis revealed 139 upregulated and 420 downregulated genes (|fold change(FC)| ≥ 1.2 and *P* < 0.05) in DIC-treated cells compared to controls. Gene expression differences were visualized in the form of a volcano plot (Figure 2(a)). Differences in biological function GO enrichment analysis based on natural processes (BPs), molecular procedures (MFs), and cellular components (CCs) were evaluated to gain insights into biological function (Figure 2(b)). Differential expression primarily affected BPs, including cellular process, natural regulation, regulation of the birth process, and metabolic process. The major MFs influenced were implicated in binding, catalytic activity, molecular function regulator, and molecular transducer activity. The primary CCs affected were cells, cell parts, and organelles. KEGG pathway analysis was further performed to identify the top 20 signaling pathways influenced by the differentially expressed genes (Figure 2(c)). The differentially expressed genes influenced the osteoclast differentiation pathway (red frame in Figure 2(c)). KEGG pathway annotation indicated that multiple genes were enriched in signal transduction and signaling molecules and interactions, confirming the KEGG enrichment findings (Figure 2(d)). A PPI network of coregulated downregulated genes was obtained and visualized (Figure 2(e)). NFATc1 had multiple connections, suggesting that it might be the key molecule involved.

### 4.3. Dictamnine Suppresses RANKL-Induced Osteoclast Formation

**Figure 3:**
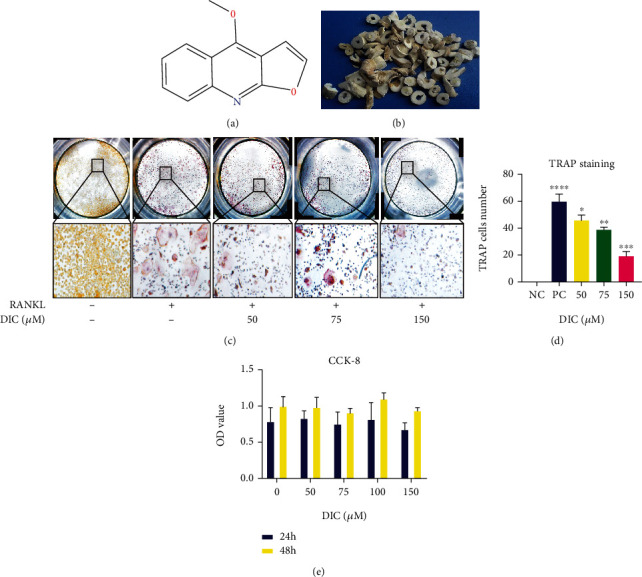
Dictamnine suppresses RANKL-induced osteoclast differentiation with no cytotoxicity in vitro. (a) The molecular formula of dictamnine. (b) Graphical representation of the source of dictamnine. (c) RAW264.7 cells seeded in a 96-well plate induced by RANKL at 50 ng/ml for 96 hours in the presence or absence of DIC. TRAP staining was performed until mature osteoclasts were produced. DIC inhibited the formation of osteoclasts in a dose-dependent manner. (d) Quantification analysis of TRAP-positive cells. (e) The cytotoxic and proliferative effects of different concentrations of DIC measured by the CCK8 method at both 24 and 48 hours. All bar charts are presented as the mean ± SD; *n* = 3; scale bar = 200 *μ*m. ^∗^*P* < 0.05, relative to the nontreatment group.

The molecular formula is shown (Figures 3(a) and 3(b)). A CCK-8 kit was used to measure cell viability and cytotoxicity. The results indicated that DIC has no toxic effect on RAW264.7 cells and does not affect cell viability. We evaluated treated cells at 24 hours and 48 hours to test whether the different concentrations of DIC (50 *μ*m, 75 *μ*m, 100 *μ*m, and 150 *μ*m) can affect the viability of RAW264.7 cells (Figure 3(e)). None of the cell viability measurements were statistically significant, indicating that the cells' independent differentiation ability formed the fundamental basis of further experiments. Meanwhile, we chose three (50 *μ*m, 75 *μ*m, and 150 *μ*m) out of the four dictamnine concentrations for the osteoclast formation assay (Figures 3(c) and 3(d)). The criterion for TRAP staining was three or more multinucleated giant osteoclasts. After 96 hours of RANKL induction, compared to the positive control, our results showed that dictamnine inhibited osteoclast formation by reducing the counts of mature osteoclasts in a dose-dependent manner. Among them, 150 *μ*m dictamnine significantly inhibited OC formation (Figure 3).

### 4.4. Dictamnine Ameliorated Osteoclastogenesis via RANKL-Activated NF-*κ*B Signaling and Downregulated Osteoclast Formation-Related Genes

**Figure 4:**
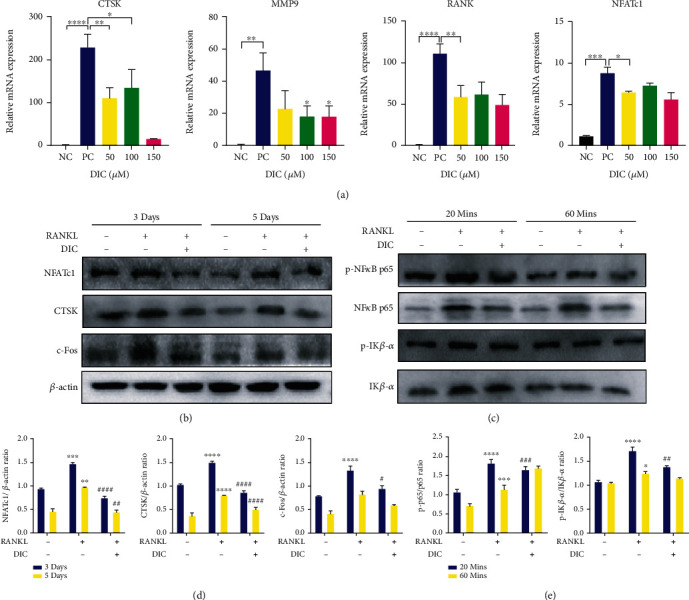
DIC abrogates NFATc1 and CTSK expression, which is further related to specific osteoclastogenesis genes, and inhibits the NF-*κ*B/I*κ*B*α* pathway (a). Expression of specific genes during osteoclast differentiation. DIC suppresses the mRNA levels of CTSK, MMP9, RANKL, and NFATc1 (b). Representative images show the inhibitory effects of DIC on NFATc1, CTSK, and c-Fos (c). Quantification of the band intensity ratios of NFATc1, CTSK, and c-Fos relative to *β*-actin (d). Western blotting was used to analyze the primary antibodies against NF-*κ*B p65/p-p65/I*κ*B*α*. The images show that DIC inhibited the NF-*κ*B pathway and promoted I*κ*B*α* downstream (e). Quantitative analyses of p-p65 were normalized to total p65. The phosphorylation levels of NF-*κ*B p65/p-p65/I*κ*B*α* were significantly suppressed by DIC from 20 minutes to 60 minutes. All bar charts are presented as the mean ± SD; *n* = 6. ^∗^*P* < 0.05, relative to the nontreatment group. ^#^*P* < 0.05, relative to the RANKL treatment group.

We attempted to probe how DIC regulates the process of osteoclast differentiation at the molecular stage. We detected the essential genes expressed explicitly in osteoclast formation. After 3 or 5 days of DIC administration and RANKL induction, the protein levels of CTSK, c-Fos, and NFATC1 were significantly decreased (Figures 4(b) and 4(d)). Meanwhile, at the mRNA expression level, the osteoclast differentiation genes RANK, NFATc1, MMP9, and CTSK showed trends of inhibition in a dose-dependent manner (Figure 4(a)). The above results indicate that DIC can reduce the protein expression of CTSK, c-Fos, and NFATC1 and downregulate specific osteoclast differentiation genes (Figure 4(d)). To perform a more in-depth elucidation of the mechanism, we investigated the related pathways of osteoclasts induced by RANKL; the results showed that the ratio of phosphorylated p65 to total p65 decreased significantly after 20 and 60 minutes of DIC treatment. The downward trend of I*κ*B*α* after DIC treatment was the same as that of NF-*κ*B. These findings indicate that DIC inhibits the signal transduction of NF-*κ*B and its downstream targets and inhibits the activity of I*κ*B*α* (Figures 4(c) and 4(e)).

### 4.5. Dictamnine Reduced the Production of the RANKL-Induced F-Actin Belt

**Figure 5:**
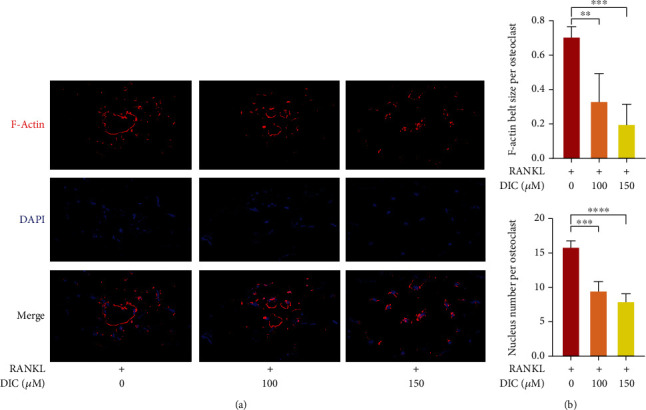
DIC attenuates RANKL-induced actin ring formation. (a) RAW264.7 cells were plated into a 96-well plate and stimulated with 20 ng/ml RANKL in the presence or absence of the indicated concentration of DIC. The cells were fixed with 4% PFA, permeabilized with 0.1% Triton X-100 solution, and stained with phalloidin (green fluorescence) and DAPI. A representative image of actin band formation was viewed with a confocal microscope, with the F-actin band stained red and the nucleus stained blue. (b) Quantitative analyses of the F-actin band and nucleus numbers per cell. All bar charts are presented as the mean ± SD; *n* = 3; ^∗^*P* < 0.05, relative to the non-DIC treatment group.

The F-actin belt is essential for bone resorption by osteoclasts; we examined whether DIC could damage the bone resorption presented by the F-actin belt. Positive F-actin belts were detected under RANKL induction, while the F-actin belt areas were markedly decreased in DIC-treated groups. Notably, the areas of the F-actin belts were reduced by DIC, indicating that precursor cell fusion was inhibited (Figure 5(a)).

### 4.6. The Level of ROS Production Was Reduced after Dictamnine Treatment

**Figure 6:**
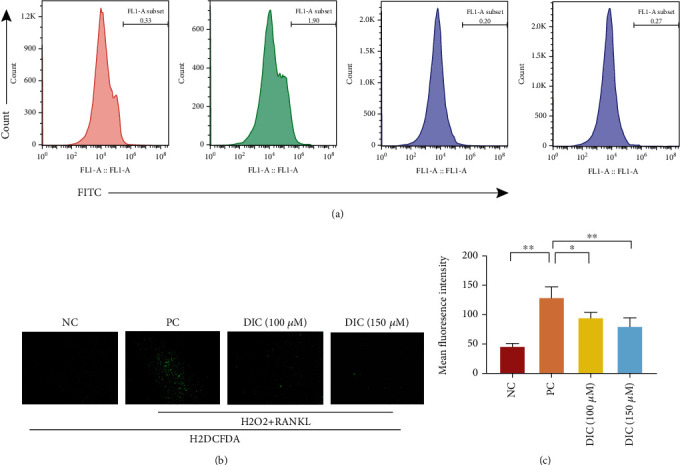
DIC reduces intracellular ROS activity. (a) Representative flow cytometry images of RANKL-induced ROS production in the presence or absence of pretreated DIC. An H2DCFDA probe detected intracellular ROS in the form of highly fluorescent DCF. (b) With or without DIC intervention, confocal images were measured using a fluorescent probe emitting a fluorescence signal similar to FITC. (c) Quantitative analyses of fluorescent intensity. All bar charts are presented as the mean ± SD; *n* = 3; ^∗^*P* < 0.05, relative to the non-DIC treatment group.

To explore the ROS levels in RAW264.7 cells, an H2DCFDA probe was used. The effect of DIC on ROS production was measured by both fluorescence microscopy (Figure 6(b)) and flow cytometry (Figure 6(a)). The results indicated that DIC significantly decreased ROS levels in RANKL-associated osteoclast formation by fluorescence microscopy and flow cytometry. Compared to the positive control, to which 3% H_2_O_2_ was added, the fluorescence intensity was weakened in the DIC-treated group. Thus, these data indicated that DIC effectively inhibited ROS production in RAW264.7 cells after RANKL induction.

### 4.7. Dictamnine Protects against Bone Loss Caused by Ovariectomy in C57BL/6J Mice

**Figure 7:**
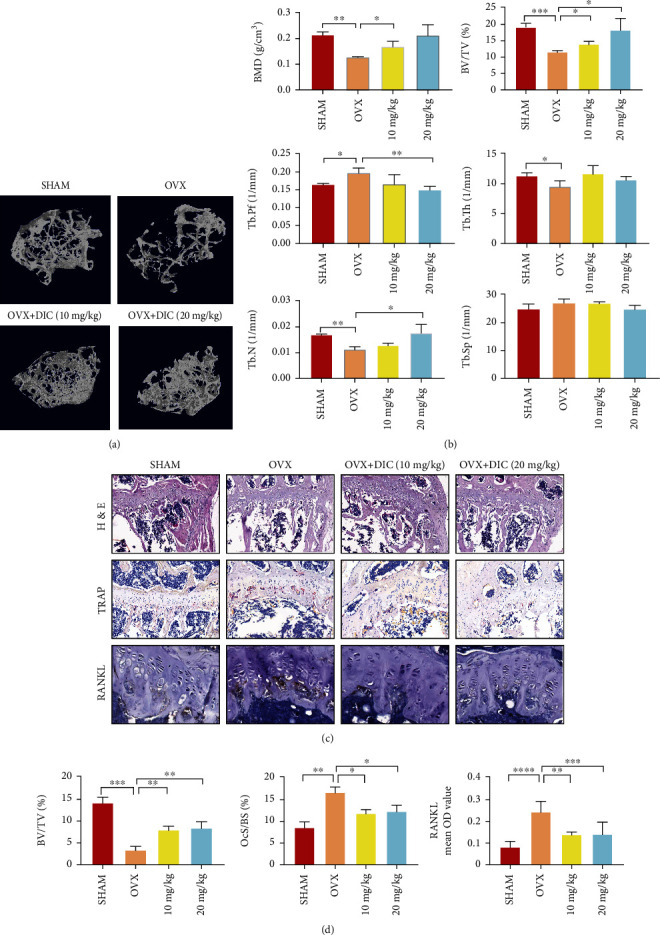
DIC prevents OVX-induced bone mass loss *in vivo*. (a) Micro-CT images of sagittal and tibial transverse views of the knee joint in different groups of mice. (b) Trabecular bone parameter analysis of BMD, BV/TV, Tb.N, Tb.Pf, Tb.Th, and Tb.Sp. (c) H&E staining indicated that the thickness of the bone trabecula surface was more maintained in the DIC-treated groups than in the OVX group. The expression of RANKL was determined by IHC staining and compared to those in the DIC-treated and sham groups. The sections in the OVX group showed a high expression trend of RANKL in a dose-dependent manner. The activity of osteoclasts was assessed by TRAP staining. (d) Quantitative analyses of HE and TRAP staining, including BV/TV (*n* = 5) and OcS/BS (*n* = 5). All bar charts are presented as the mean ± SD. ^∗^*P* < 0.05, ^∗∗^*P* < 0.01 compared with the OVX group.

Based on the cellular results, we established the ovariectomy mouse model because it is a classical model that can explore the effect of PMOP. The bilateral knee joint CT scan verified that the trabecular BMD, Tb.Th, and Tb.N of the OVX group were reduced primarily to the sham group, indicating successful model establishment [23]. The ratio of bone volume to tissue volume (BV/TV), which can directly reflect the change in bone mass, is also an indirect index of bone mass; our results revealed the same tendency as BMD. Administration of DIC markedly protected the animals from OVX-associated bone loss and trabecular deterioration both in the low- and high-dose groups. The BMD, BV/TV, number, thickness, and separation of trabeculae (Tb.N, Tb.Th, and Tb.Sp) recovered after DIC administration (Figure 7(a)). It is worth noting that the trabecular pattern factor increased in the DIC groups, indicating that the trabecular bone changed from the plate to the rod. H&E staining revealed that the bone volume and surface in OVX mice were well preserved after DIC treatment compared to those in the control group. The results of IHC staining showed that DIC had the opposite effect on the expression of RANKL in the trabeculae of tibial tissues. In contrast, TRAP staining indicated that DIC decreased the activity of osteoclasts in OVX mice (Figure 7(b)). The quantification of DIC retarded bone resorption, which led to the protective effect of OVX-associated bone loss.

## 5. Discussion

PMOP is characterized by estrogen deficiency, in which the rapidity of bone resorption becomes faster than that of bone formation, and bone loss becomes accelerated [24]. Osteoporosis is recognized by the occurrence of low–energic traumatic fractures. The incidence rate of fractures is lower in young females than in young males, but it increases sharply above 35 until the female's morbidity rate becomes twice that of males. PMOP imposes a substantial economic burden and causes high incidence and mortality [25].

Multidisciplinary approaches are needed to prevent osteoporosis, including estrogen replacement therapy during perimenopause, physical exercises, and modified eating and living habits [26]. The prevention of PMOP is long term; the use of a native compound may become a suitable method. In this study, we concluded that dictamnine inhibits osteoclastogenesis by inhibiting the activities of ROS, NF-*κ*B, and NFATc1 in vitro and deters the development of OVX-induced osteoporosis mouse models.

Recent studies have found that other compounds also alleviate osteoclastogenesis and ovariectomized osteoporosis by inhibiting RANKL-induced activation of ROS and NFATc1 at relatively low doses [27, 28]. Dictamnine (DIC) was used at relative high doses at 150 *μ*M in vitro and 10–20 mg in vivo. This concentration of DIC mainly resists osteoporosis through this pathway, perhaps, different concentrations may produce other biological behaviors, and also, we are exploring whether different concentrations may resist bone loss in other pathways.

We established a crystal model predicting the combination of DIC and RANKL, and a strong affinity between them was observed in computational docking. This provided preliminary evidence that DIC may play a role in binding RANKL. Furthermore, RNA sequencing was performed and the differentially expressed genes and their bioinformatics analysis showed an important impact on the osteoclast differentiation pathway. This further suggests that DIC may suppress osteoclast formation by binding RANKL.

High expression of TRAPase is characteristic of multinucleated osteoblasts [29]. To evaluate the biological function of DIC, osteoclast differentiation induced by DIC was measured. In the DIC-treated group, the number of osteoclasts was significantly reduced in a dose-dependent manner.

We further explored the mechanism by which DIC inhibits osteoclast formation. Cathepsin k (CTSK), secreted by osteoclasts, can degrade collagen and matrix protein during bone resorption [30]. Activation protein 1 (ap-1, c-Fos) stimulates CTSK activity and activates NFATc1. The results showed that both c-Fos and NFATc1 could stimulate the promoter activity of CTSK alone, and the combined effect of c-Fos and NFATc1 can maximize the movement of CTSK [31–33]. NFATc1, a key transcription factor, can activate downstream genes with its nuclear translocation. The lack of NFATc1 makes embryonic stem cells unable to form osteoclasts. The destruction of NFATc1 in hematopoietic cells led to decreased osteoclasts and increased bone mass in a mouse model [34–36]. Our results show that the expression level of CTSK is significantly inhibited by DIC, and the activity levels of its activator protein c-Fos and the classical transcription factor NFATc1 are also considerably suppressed. Other osteoclast-specific genes, such as MMP9, are also regulated by DIC.

The physiological function of osteoclasts is to degrade the bone matrix, while the matrix is synthesized with collagen I extracellular matrix (ECM). The F-actin rings are identified as supplying a cavity corresponding to the resorption dot by directing osteoclasts to the mineral matrix [37]. Therefore, the essential element of osteoclast resorption is F-actin [38]. Interestingly, in the DIC-treated group, the rings seemed to be smaller but were still attached to each other but the number of nuclei in each osteoclast did not decrease. This further demonstrates the biological function of DIC in inhibiting osteoclast formation.

ROS can act synergistically with osteoclasts. The ROS produced by osteoclasts regulate bone homeostasis by stimulating and promoting the resorption of bone tissues [13]. Higher ROS levels indicate more vital bone resorption ability of osteoclasts. In a deeper exploration of the mechanism, DIC also affects the activity of osteoclasts by regulating ROS. Studies have shown that ROS act as mediators in RANKL-induced osteoclast differentiation, such as controlling the movement of the vital osteoclast transcription factor NF-*κ*B [39]. ROS can regulate NF-*κ*B activation by inhibiting the phosphorylation of I*κ*B*α* [39, 40]. NFATc1, another key osteoclast transcription factor, is also related to ROS activity. A few days after the initial activation of NFATc1, other signals from persistent Ca2+ oscillations will trigger the automatic amplification of NFATc1, which is essential for increasing gene transcription to drive precursor fusion and maturation [10]. RANKL also induces Ca^2+^ oscillations, leading to the upregulation and automatic amplification of NFATc1. In the above study, we demonstrated that DIC inhibited the transcriptional activity of NFATc1 and interfered with the expression of NF-*κ*B p65 and its phosphorylated form, thereby reducing the expression of I*κ*B*α* [41, 42]. However, we can only verify that DIC regulated ROS levels, downregulated the NF-*κ*B/I*κ*B*α* pathway, and inhibited the production of NFATc1. Whether this biological process depends on the ROS level remains to be further verified.

As OVX closely mimics the characteristics of bone changes associated with postmenopausal osteoporosis, OVX mice are widely used to study bone resorption caused by estrogen deficiency. Mouse models of ovariectomy were established to confirm that DIC can be a potential drug for treating osteoporosis *in vivo*. Micro-CT scanning revealed that both BMD and BV/TV were improved. The number of bone trabeculae shown by HE staining was well preserved compared with that in the model group. These results suggest that DIC can inhibit osteoclast production and play a role in protecting bone loss *in vivo*. The formation of osteoclasts is closely related to RANKL, and OPN, OCN, and ALP are essential genes in bone formation [43]. Immunohistochemical results indicated that RANKL expression in the model group was significantly increased in the model group compared to the sham group. Nevertheless, it was significantly decreased after DIC administration. The expression levels of other bone formation genes increased significantly after DIC administration. These results show that DIC can promote the expression of crucial proteins in bone formation and inhibit the expression of RANKL in bone tissue.

Our present study's explicit limitation is the lack of an in vitro intracellular calcium oscillation test and the ability to quantify the biomechanical properties of the bone in the mouse OVX model, which would allow us to dissect intracellular Ca2+ levels related to the protein synthesis and the biomechanical properties of bone in the DIC-treated OVX model. Therefore, future studies are needed to verify the combination of DIC and RANKL at the computational and molecular levels.

**Figure 8:**
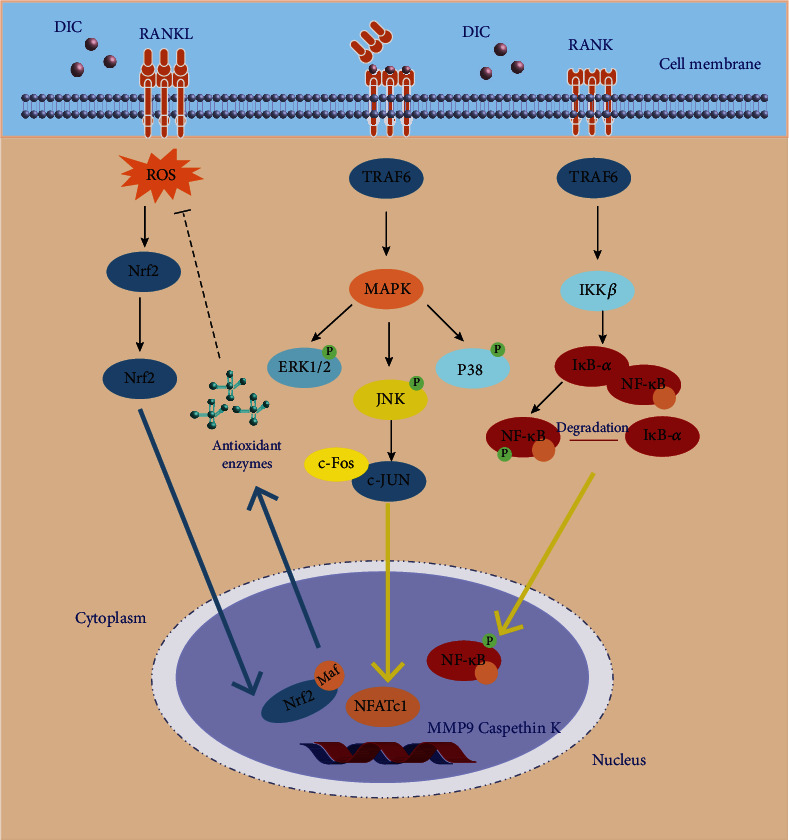
The process and mechanism by which DIC can inhibit osteoclast formation. Schematic diagram of the molecular regulation of DIC RANKL-induced osteoclastogenesis. DIC leads to attenuation of nuclear translocation and autoamplification of NFATc1 through inhibition of RANKL and RANK binding, MAPK and NF-*κ*B pathways, and blockade of calcium oscillations. The production of reactive oxygen species (ROS) and scavenging of ROS by antioxidant enzymes are regulated by DIC. GTP: guanosine-5′-triphosphate; MAPK: mitogen-activated protein kinase; JNK: c-Jun N-terminal kinase; TRAF6: TNF receptor-associated factor 6; Erk: extracellular signal-regulated kinase; NFATc1: nuclear factor 1 of activated T cells.

In conclusion, our study proves that DIC, a novel furoquinoline alkaloid, can inhibit the formation and function of osteoclasts by inhibiting ROS levels and F-actin ring production. Osteoclast formation was affected by downregulating CTSK and NFATc1 osteoclast differentiation-specific genes, which was achieved by significantly inhibiting the production of the NF-*κ*B/I*κ*B*α* signaling pathway (Figure 8). DIC also inhibits the expression of RANKL in bone tissue, promotes the expression of OPN and ALP, and prevents OVX-induced osteoporosis *in vivo*. In summary, these findings pave the way for the targeting of DIC for bone diseases such as osteoporosis.

## Figures and Tables

**Table 1:** The primers of each factor.

MMP9:	F:5′-GCAGAGGCATACTTGTACCG-3′
	R:5′-TGATGTTATGATGGTCCCACTTG-3′

CTSK:	F:5′-CTCGGCGTTTAATTTGGGAGA-3′
	R:5′-TCGAGAGGGAGGTATTCTGAGT-3′

RANK:	F: 5′-TCCTCGGGAACTGGCTATG-3′
	R:5′-GGTTGGGTCCCATTGGAGAC-3′

NFATC1:	F: 5′-GGAGAGTCCGAGAATCGAGAT-3′
	R:5′-TTGCAGCTAGGAAGTACGTCT-3′

## Data Availability

All data generated and analyzed in the present study are available from the corresponding author upon reasonable request.
